# Modelling the Impact and Cost-Effectiveness of Biomarker Tests as Compared with Pathogen-Specific Diagnostics in the Management of Undifferentiated Fever in Remote Tropical Settings

**DOI:** 10.1371/journal.pone.0152420

**Published:** 2016-03-30

**Authors:** Yoel Lubell, Thomas Althaus, Stuart D. Blacksell, Daniel H. Paris, Mayfong Mayxay, Wirichada Pan-Ngum, Lisa J. White, Nicholas P. J. Day, Paul N. Newton

**Affiliations:** 1 Mahidol-Oxford Tropical Medicine Research Unit (MORU), Faculty of Tropical Medicine, Mahidol University, Bangkok, Thailand; 2 Centre for Tropical Medicine and Global Health, Nuffield Department of Medicine, University of Oxford, Oxford, United Kingdom; 3 Lao-Oxford-Mahosot Hospital-Wellcome Trust Research Unit (LOMWRU), Mahosot Hospital, Vientiane, Laos; 4 Faculty of Postgraduate Studies, University of Health Sciences, Vientiane, Laos; Instituto de Higiene e Medicina Tropical, PORTUGAL

## Abstract

**Background:**

Malaria accounts for a small fraction of febrile cases in increasingly large areas of the malaria endemic world. Point-of-care tests to improve the management of non-malarial fevers appropriate for primary care are few, consisting of either diagnostic tests for specific pathogens or testing for biomarkers of host response that indicate whether antibiotics might be required. The impact and cost-effectiveness of these approaches are relatively unexplored and methods to do so are not well-developed.

**Methods:**

We model the ability of dengue and scrub typhus rapid tests to inform antibiotic treatment, as compared with testing for elevated C-Reactive Protein (CRP), a biomarker of host-inflammation. Using data on causes of fever in rural Laos, we estimate the proportion of outpatients that would be correctly classified as requiring an antibiotic and the likely cost-effectiveness of the approaches.

**Results:**

Use of either pathogen-specific test slightly increased the proportion of patients correctly classified as requiring antibiotics. CRP testing was consistently superior to the pathogen-specific tests, despite heterogeneity in causes of fever. All testing strategies are likely to result in higher average costs, but only the scrub typhus and CRP tests are likely to be cost-effective when considering direct health benefits, with median cost per disability adjusted life year averted of approximately $48 USD and $94 USD, respectively.

**Conclusions:**

Testing for viral infections is unlikely to be cost-effective when considering only direct health benefits to patients. Testing for prevalent bacterial pathogens can be cost-effective, having the benefit of informing not only whether treatment is required, but also as to the most appropriate antibiotic; this advantage, however, varies widely in response to heterogeneity in causes of fever. Testing for biomarkers of host inflammation is likely to be consistently cost-effective despite high heterogeneity, and can also offer substantial reductions in over-use of antimicrobials in viral infections.

## Introduction

Declining malaria transmission and increasing availability of malaria rapid diagnostic tests (RDTs) together suggest that the majority of febrile patients are identified as having non-malarial causes of fever [1, 2]. For these patients, health workers in remote settings currently rely at best on syndromic algorithms to determine whether treatment with antibiotics is required. These algorithms, however, are of limited accuracy, resulting in over-prescription of antibiotics in viral infections, while bacterial infections often go untreated [3, 4].

One approach to improve the management of non-malarial fevers is the use of further pathogen-specific diagnostics to identify the aetiology. This can inform as to whether antibiotics are required, and for bacterial pathogens as to the most appropriate treatment. Such rapid tests are increasingly available, although with the exception of those for influenza and dengue, few have demonstrated sufficient accuracy for use at point-of-care [5, 6]. As more such tests become available, these could be used alone or in combination to identify causes of illness.

There are, however, limitations to this approach. Optimising the configuration of multiple possible tests poses logistical and economic challenges in the context of extensive epidemiological, seasonal and spatial heterogeneity characteristic of the rural tropics. Furthermore, the tests themselves might require adaptation to local context. For example, scrub typhus RDTs detecting antibody might require adaptation of their positivity cut-off thresholds with reference to the population background antibody titres to optimize their diagnostic accuracy [7].

An alternative approach is testing for biomarkers of host inflammation. While inflammation can result from a broad range of infectious and non-infectious causes, some biomarkers have been shown to discriminate well between infections that need antibiotics and those that require only symptomatic care [8, 9]. Biomarkers that have been extensively studied in primary, emergency, and critical care include C-reactive protein (CRP) and procalcitonin [10–13]. In the largest study of these biomarkers in a tropical, Southeast Asian context, CRP was found to outperform procalcitonin in its ability to discriminate between viral and bacterial infections in well-characterised samples from febrile patients [14]. Inexpensive and accurate CRP rapid diagnostic tests are already commercially available [15, 16].

Evaluations of pathogen-specific and biomarker tests have largely focused on their diagnostic accuracy. How they are incorporated into patient management guidelines and health worker decision-making, however, will determine their actual impact on patient treatment and health outcomes. For instance, should all patients with a negative dengue or influenza test be prescribed an antibiotic? In areas where scrub typhus is known to be a frequent treatable infection, is a negative scrub typhus test sufficient to discourage antibiotic prescription? If CRP testing is implemented, what are the most relevant thresholds to inform the need for an antibiotic?

Potential improvement in antibiotic targeting also depends on current empirical treatment practices. In areas where treatment is less readily available, the greatest benefit of introducing new tests might be in ensuring that patients requiring antibiotics are identified as such. The tangible, direct health benefits of this are measurable and can be readily weighed against the higher costs of testing. In other contexts where prescription rates are already high, the benefit of introducing new diagnostics might primarily be in reducing unnecessary antibiotic consumption. While the societal benefits of these gains are intuitively clear, they are far more difficult to quantify for cost-benefit evaluations [17].

Even when a patient is correctly identified as requiring an antibiotic, the choice of antibiotic will also be important in ensuring effective treatment. Here too the utility of the testing approaches will vary based on the relevance of empirical treatment guidelines. For example, in areas where bacterial infections such as leptospirosis and/or rickettsioses are prevalent but guidelines do not recommend the use of a tetracycline antibiotic, there is greater benefit for tests that identify these pathogens to inform on the use of an appropriate antibiotic than in areas where these antibiotics are already widely used.

In this study we compare the approaches of using pathogen-specific diagnostics and biomarker tests in undifferentiated fever in a rural Laos setting. This is not an attempt to determine which approach or test is definitively superior–this will vary widely, dependent on many factors such as the accuracy of the tests, local aetiologies of fever, and the value policy makers place on avoiding unnecessary antimicrobial consumption. The aims here are to identify broad trends in the ability of the two approaches to improve antibiotic targeting in primary care and produce preliminary indications of how cost-effective they might be as compared with current practice in community care of febrile patients in the rural tropics.

## Methods

### Patient characteristics

We used data from a fever study carried out in two sites in rural Laos, where causes of fever were investigated in a total of 1938 patients, focusing here on the 1083 outpatients, the population in which we were interested in modelling use of the tests [18]; aetiologies and prognoses in inpatients with more severe disease could be different requiring other diagnostic and treatment strategies than those modelled here. A probable single microbiological cause of illness was identified in 33% of the outpatients.

The first aim of the analysis was to compare the ability of health workers’ to decide when an antibiotic was required in the original study (on presentation, without knowledge of the final diagnosis) [18], with the two approaches of using a pathogen-specific test or testing for a biomarker of inflammation. For the pathogen-specific approach, we modelled use of rapid tests for dengue and for scrub typhus. A dengue test could be informative given the relatively high incidence of dengue in the region [1]; several validated dengue tests are already commercially available [19]. Scrub typhus rapid tests are commercially available and could help diagnose what is emerging as a key important treatable infection in the Mekong region [7]. For the biomarker approach, we modelled use of a CRP rapid test, based on evidence supporting its ability to distinguish bacterial from viral infections prevalent in Southeast Asia [14]. Rapid qualitative and semi-quantitative lateral flow tests are commercially available and some have been shown to accurately detect elevated CRP levels as compared with quantitative readers [20].

### Test characteristics

Several dengue RDTs have been available for a number of years with reported accuracy varying widely [21]. Here we assumed a sensitivity and specificity of 95% for a dengue RDT when performed on presentation, as might be achieved with a combined IgM/IgG plus NS1 rapid test. Scrub typhus IgM RDTs are increasingly available but not as well validated [22]; for the purposes of this model we assumed the same baseline accuracy with no cross reactivity with other rickettsial infections. For the CRP RDT we used the CRP levels measured in these patients with a threshold of 20mg/L as an indication of requiring antibiotics [10].

### Patient management algorithms

**Fig 1:**
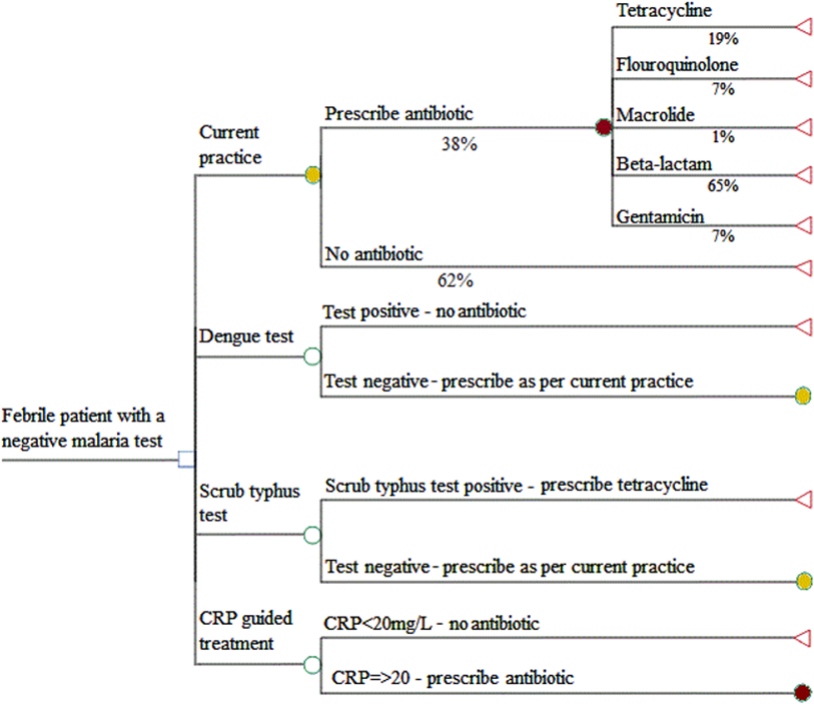
Algorithms for antibiotic prescription for each of the strategies. The yellow circular nodes in the pathogen-specific branches replicate current practice in terms of whether an antibiotic is prescribed and the choice of antibiotic. Patients with elevated CRP receive an antibiotic, with the choice of drug reflecting current practice.

We assumed the following algorithms would inform how the test results would be used to inform treatment practices (Fig 1):

- Patients with a positive dengue test are not prescribed an antibiotic
- Patients with a positive scrub typhus result are prescribed an effective antibiotic (tetracycline or macrolide)
- Where a pathogen-specific test is negative, antibiotics are prescribed at random at a rate of 38%, with the choice of antibiotic resembling current practice as observed in the fever study
- Patients with CRP levels below the threshold of 20mg/L do not receive an antibiotic, and those above the threshold do, with the choice of antibiotic resembling current practice.

### Modelling antibiotic targeting

The proportion of patients with a bacterial infection that received antibiotics and those with viral infections in whom antibiotics were not prescribed was calculated using the incidence of each pathogen, the test result, and following the above algorithms. We then explored the robustness to heterogeneity and uncertainty in causes of fever and to the assumed test accuracies by varying the incidence of infections using uniform distributions ranging from half to double the incidence in the fever study and applying beta distributions to the assumed accuracies of the tests, as described in Table 1. The antibiotic prescription rate in patients with a negative test for dengue or scrub was also selected at random from a uniform distribution between 0 and 100%. We carried out 500 Monte Carlo simulations, sampling from these distributions to calculate the total proportion of patients in whom antibiotics were prescribed correctly (i.e. antibiotics prescribed in bacterial infections and not in viral infections).

**Table 1:** Model parameters and probability distributions in the probabilistic sensitivity analyses.

Parameter	Point estimate	Probability distribution	Notes/references
Sensitivity and specificity of dengue and scrub typhus tests	95%	Beta (α = 47.5,β = 2.5)	These estimates are mostly higher than documented in the literature, assuming continual improvement in their quality [22, 24–26].
Mortality rate for bacterial infections in the absence of an effective antibiotic	1%	Beta (α = 49.5,β = 0.5)	Data for this are scarce for ethical reasons. We use a Delphi survey result indicating that 20% of non-malarial fevers in adults in the tropics are of bacterial origin, and left untreated 20% of these will become severe; these severe cases are associated with a 30% mortality rate [27] [28].
Years of life lost per death	45	One way sensitivity analyses (20–60)	Based on the median age of outpatient and life expectancy at birth in Laos [29]
Cost of RDTs	$1.5	Gamma (shape = 6, scale = 0.25)	RDTs for dengue and scrub that have been validated in the literature are of higher cost. It is assumed however that as with malaria RDTs these will come down in price if used on a larger scale. Low cost (<$1) RDTs for dengue and CRP are already commercially available but few have been evaluated.
Cost of a course of antibiotics	$0.5	Gamma(shape = 2, scale = 0.25)	[23]
Probability of treatment in patients with a negative dengue or scrub typhus test	38%	Uniform (0%-100%)	This determines the trade-off between under/over-treatment in the absence of a diagnosis or biomarker test and will vary widely by setting [30–32]

We then extend the analysis to address the more nuanced question of how well antibiotics are targeted in bacterial infections given their susceptibility to the different drugs. For this we drew on the antibiotic prescription data in the fever study and considered for each pathogen the probability of being prescribed a specific antibiotic, and the probability that this antibiotic would be effective for this pathogen. These data suggest that current empirical treatment practices are most often inadequate, with the probability of receiving *an effective* antibiotic in treated patients ranging from 20% in patients with scrub typhus (usually treated with tetracyclines or macrolides), through 56% for those with leptospirosis, to 82% for those with bacteraemia [18]. Other than for patients with a positive scrub typhus result whom are assumed to receive doxycycline, we assumed that the choice of antibiotics would reflect these current, often inadequate empirical treatment practices.

### Cost-effectiveness analysis

We performed a rudimentary cost-effectiveness assessment of the tests in the context of primary care. Given the extensive uncertainty in most parameters we performed a probabilistic sensitivity analysis (PSA) with relevant distributions as described in Table 1. For the costing we assumed that the differences in resource use are only those related to the diagnostics and treatments. Other capital and labour overheads were assumed to be similar in all strategies. The costs for point-of-care tests are not well described therefore we applied a gamma distribution with an assumed mean of $1.5, slightly higher than current malaria RDTs. The cost of a course of antibiotic is estimated at a mean of $0.5 [23].

For health outcomes, we assumed all self-limiting viral infections and treated bacterial infection are associated with a week of ill health with a disability weight of 0.053, as estimated in the 2010 Global Burden of Diseases for an acute, moderate, episode of infectious disease [33]. We assumed that bacterial infections that do not receive an appropriate treatment are associated with a further week of illness and a 1% mortality rate [27]; each of these deaths is associated with a mean loss of 45 life-years (the median age of outpatients was 20 and Laos PDR has a life expectancy of 65 years).

For strategies that are more costly and more effective than current practice we calculated the median incremental cost per disability adjusted life year (DALY) averted and report the 2.5%-97.5% credible interval (CrI). We assumed a willingness to pay (WTP) threshold to classify interventions as cost-effective of $1400, approximating Laos 2012 GDP per capita [29]. We also produced cost-effectiveness acceptability curves that illustrate the probability of each strategy being cost-effective as compared with current practice across a broader range of WTP thresholds.

### Ethical approval

The analyses draw on microbiological investigations and CRP assays carried out in previous studies for which ethical approval was provided by the Lao National Ethics Committee for Health Research and from the Oxford Tropical Research Ethics Committee (OXTREC). These approvals included evaluation of diagnostics tests and biomarkers of host inflammation as performed in this analysis. All patient records/information was anonymized and de-identified prior to analysis.

The model and outputs were produced using R 3.2.1. and RStudio (Integrated Development for R. RStudio, Inc., Boston, MA URL http://www.rstudio.com/).

## Results

**Fig 2:**
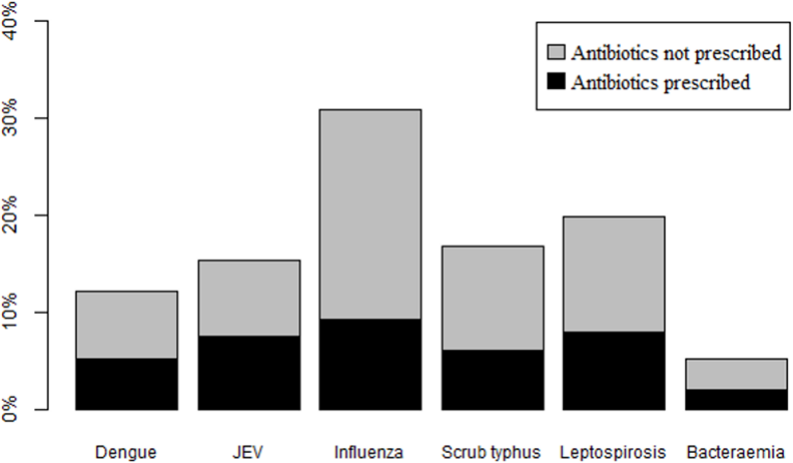
Percentage of microbiologically confirmed diagnoses out of all outpatients, and of those the proportion prescribed an antibiotic. JEV–Japanese encephalitis virus.

Of the 1083 outpatients recruited into the fever study in Laos, 353 (33%) patients had a microbiologically confirmed diagnosis. A total of 58% of these had a viral infection and 42% had a bacterial infection. Influenza was the most common microbiologically confirmed cause of illness (31%), followed by leptospirosis (20%) and scrub typhus (15%). Overall 38% of outpatients received an antibiotic with no indication of these being concentrated in patients with a bacterial infection. The aetiological data and the proportion of patients prescribed an antibiotic are shown in Fig 2.

Table 2 shows the percentage of infections that according to the model would be prescribed an antibiotic based on the estimated test accuracies and algorithms, as compared with current practice as documented in the original fever study.

**Table 2:** Percentage of patients with each infection that would receive an antibiotic using either a dengue RDT, a scrub typhus RDT or CRP RDT with a threshold of 20mg/L, as compared with current practice.

	Aetiology	Current practice	Dengue RDT	Scrub typhus	CRP 20mg/L
**Antibiotics not beneficial**	Dengue	43%	4%	45%	20%
	Japanese encephalitis virus infection	49%	35%	45%	32%
	Influenza	30%	35%	45%	18%
**Antibiotics beneficial**	Scrub typhus	36%	35%	90%	75%
	Leptospirosis	40%	35%	45%	89%
	Bacteraemia	40%	35%	45%	89%

**Fig 3:**
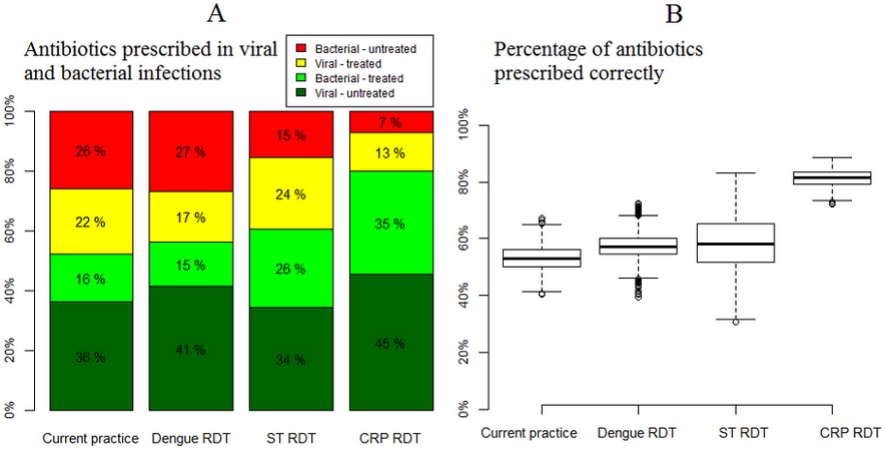
**Antibiotic targeting using the Laos data (A) and across a range of simulated incidences and test accuracies (B).** Figure A illustrates that CRP testing achieved the largest proportion of patients that are correctly prescribed an antibiotic (the bottom dark green segment of the bars show the percentages of patients with viral infections not prescribed an antibiotic; the light green segment shows the percentages of patients with bacterial infections correctly prescribed an antibiotic; the yellow segment shows the percentages of patients with viral infections prescribed an antibiotic; and the red segment shows the percentages of patients with untreated bacterial infections). The panel on the left shows that this advantage of CRP testing is consistent when modelling extensive heterogeneity in causes of fever, while the pathogen specific tests and the scrub typhus one in particular are more affected by such heterogeneity. ST–scrub typhus; CRP–C reactive protein; RDT–rapid diagnostic test.

When aggregating pathogens into bacterial and viral groups, the model predicted that use of either pathogen-specific tests offers modest improvements over current practice in their ability to classify patients as requiring an antibiotic. Use of dengue RDTs would lead to a reduction in antibiotics prescribed to viral infections, while use of scrub typhus RDTs implied a larger proportion of bacterial infections receiving antibiotics (Fig 3A). The CRP test outperformed the pathogen-specific tests, with reductions in both unnecessary use of antibiotics in viral infections as well as less bacterial infections going untreated. In total, 80% of patients were classified correctly using the CRP test, compared with 52% based on current practice, 46% using a dengue test, and 59% using scrub typhus test.

When varying the incidence of dengue and scrub typhus at a range of 50–200% of the fever study data, the proportion of patients correctly treated varied from 51 to 63% with the dengue test and from 54% to 64% with the scrub typhus RDT. In both instances the CRP test performed consistently better with 78–80% of patients classified correctly in terms of whether they required treatment. This advantage of CRP was robust to variation in all model parameters in the PSA, including the incidence of all infections, baseline antibiotic prescription practices, and when accounting for the uncertainty surrounding the accuracy of the tests (left hand panel of Fig 3B).

We then incorporated the probability of an antibiotic being effective for the bacterial pathogens, as well as the estimated excess duration of illness and mortality in patients that did not receive an effective antibiotic to calculate the number of DALYs averted for each strategy. The median cost per patient in current practice was estimated at $0.17 and each episode was estimated as being associated with a median of 0.08 DALYs. The model outputs indicate that the dengue testing strategy in most instances offers little or no advantage over current practice. The scrub typhus averted an average 0.031 DALYs per febrile episode (Crl 0.015; 0.386). The CRP test averted 0.017 DALYs (CrI 0.001; 0.157). The median incremental costs and DALYs averted by each of the tests as compared with current practice are shown in Table 3.

**Table 3:** Incremental cost, disability adjusted life years (DALYs) and incremental cost-effectiveness ratios (ICER) of the testing strategies as compared with current practice characterised by a median cost of $0.17 and 0.08 DALYs per episode.

	Dengue RDT	Scrub typhus RDT	CRP RDT
**Median incremental cost (CrI)**	**$1.5 (0.5; 3.2)**	**$1.5 (0.6; 3.7)**	**$1.6 (0.63; 2.95)**
**Median DALYs averted (CrI)**	**-0.006 (-0.301; 0.089)**	**0.031 (0.015; 0.386)**	**0.017 (0.001; 0.157)**
**Median ICER (CrI)**	**Dominated**	**$48 (1.5; 247)**	**$94 (4.1; 2950)**

**Fig 4:**
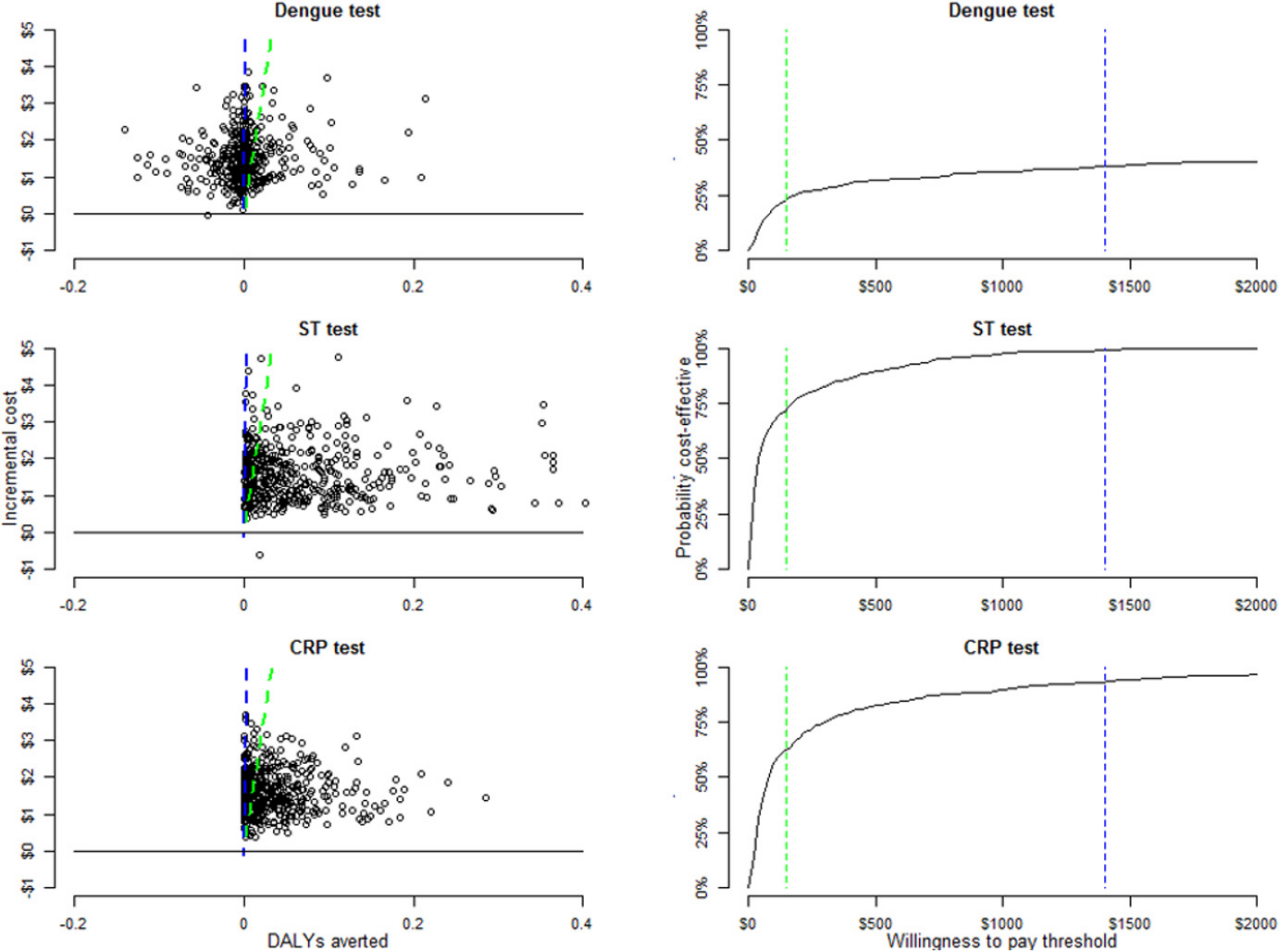
Cost-effectiveness plane and cost-effectiveness acceptability curves for the three strategies when compared with a baseline of current practice. The blue lines indicate a willingness to pay threshold of $1400, approximating the Laos GDP/capita, while the green line is a conservative willingness to pay threshold of $150. The Dengue test in most instances was associated with little or no advantage in terms of health outcomes while resulting in higher costs than current practice. The scrub typhus and CRP tests offered direct health benefits over current practice, but also at a higher cost. When accounting for parameter uncertainty, the scrub typhus and CRP tests are approximately 90% and 80% likely to be cost-effective at a willingness to pay threshold of $1400. ST–scrub typhus; CRP–C reactive protein; RDT–rapid diagnostic test.

A broad estimate for the cost per DALY averted for the three tests suggests that either the scrub typhus or CRP testing is likely to be cost-effective even at low WTP thresholds (Fig 4), despite the considerable uncertainty surrounding many model parameters.

## Discussion

The introduction of malaria RDTs overturned the decades-old practice of presumptively treating undifferentiated fevers in endemic settings with antimalarial drugs. In the Greater Mekong Subregion and many other areas in the rural tropics there are few if any tools to manage the vast majority of febrile patients who do not have malaria. Empirical antibiotic treatment is often characterised by their overuse in viral infections while patients with bacterial infections often go untreated or receive an inappropriate antibiotic.

This analysis suggests that tools that are already available can vastly improve patient management. The model predicts that pathogen-specific tests for treatable and prevalent infections such as scrub typhus in the Mekong region could offer a cost-effective strategy over current practice when considering direct health benefits to the patients. This is particularly advantageous when empirical treatment practices are ill-suited to local aetiologies, as appears to be the case in Laos [18, 34].

Accurate tests for bacterial infections however are few. Leptospirosis was a common cause of illness in this setting but rapid tests for the diagnosis of leptospirosis are of poor accuracy and unlikely to be cost-effective [35]. Typhoid tests so far have been found to be of low sensitivity in routine community care, although could be used in the context of epidemics where pre-test probabilities are higher [36]; the combination of blood culture amplification with an antigen rapid diagnostic test has been proved to be an accurate and inexpensive tool for the accelerated diagnosis of patients with typhoid [37]. Scrub typhus RDTs hold some promise but their evaluation is challenged by continued problems with defining the accuracy of the serological gold-standard [38]. This becomes increasingly problematic when aiming for high sensitivity using lower thresholds, as these are not able to distinguish active infection from immunity.

Testing for viral pathogens for which there are no immediate treatment implications such as dengue, might not offer direct health benefits over current practice and therefore this was not predicted to be cost-effective. This result, however, was highly sensitive to the antibiotic prescription rate in patients with a negative dengue test result. If all these patients were to be prescribed an antibiotic there would be a higher probability of bacterial infections receiving appropriate treatment, at the expense of high rates of over-treatment in common (non-dengue) viral infections. Furthermore, while use of dengue or influenza RDTs might not be cost-effective when considering only the health benefits for patients with bacterial infections appropriately treated, there might be other considerations in favour of their use such as patient reassurance in having a confirmed diagnosis and raising their awareness for possible danger signs, as well as alerting authorities of emerging epidemics or initiating measures for vector control.

In this analysis we focused on use of pathogen-specific tests in isolation. Another option is use of two or more tests in combination or in sequence depending on the expected rank of pathogens for the season. Pragmatically, the higher cost and logistical requirements for ensuring the supply of numerous tests, and possible confusion in the interpretation of multiple results are possible obstacles to this approach; the sophistication and number of possible algorithms to determine how results are used to inform treatment will increase exponentially with each additional (imperfect) test, and although important to consider, will suffer from a complexity that is likely to negate their public health effectiveness in rural Asia.

All pathogen-specific tests, whether used alone or in combination, suffer from a disadvantage in terms of their variable utility subject to seasonal and spatial heterogeneity, therefore to ensure their efficient use it will be important to evaluate their cost-effectiveness in relation to these contextual factors. The logistical feasibility of implementing different configurations of pathogen-specific tests by location and time of year will also need to be considered.

Testing for biomarkers of inflammation could offer a robust approach to targeting antibiotics in rural settings for heterogeneous causes of fever. In this analysis we focus on CRP testing given the large body of evidence supporting its use to guide antibiotics in acute respiratory infections and fevers [10, 14], and the commercial availability of accurate rapid tests [20]. CRP testing was consistently predicted to be the best performer in identifying whether or not antibiotics were at all required, despite heterogeneity in causes of fever, with the disadvantage of lacking indication as to the specific pathogen and therefore the optimal antibiotic. Despite the absence of this information, the ability to identify a broader range of aetiologies as requiring an antibiotic implied a consistent advantage over the other approaches. Better surveillance of aetiology of fever and antimicrobial susceptibility could inform the choice of antibiotics in whom they are indicated.

### Limitations

These simulations use the relative distribution of microbiologically confirmed cases from a fever study in Laos. These, however, represent under half of all patients recruited into the fever study, most of whom did not have an identifiable pathogen, and some of whom had multiple pathogens identified that could have been the cause of illness. A major question remains as to how well these outputs generalise to the broader population of febrile patients. It is a reasonable assumption that in cases without an identified pathogen CRP remains an effective (albeit imperfect) guide to antibiotic targeting. With the pathogen-specific tests however the implications for patients without a known diagnosis are less clear. Similarly for patients with multiple pathogens a test for a specific viral pathogen could mistakenly suggest that no antibiotic is required, when in fact a treatable bacterial infection is also present. Ultimately, however, whether either approach is effective in this broader population can and should be explored in clinical outcome studies.

Another major limitation of the economic evaluation is that it did not account for the longer term societal health and economic costs associated with extensive antibiotic consumption and subsequent resistance. Methods to incorporate these aspects into economic evaluation of diagnostics, treatments and vaccines for infectious diseases are challenging but much needed for more comprehensive assessments of the costs and benefits involved [39]. The inclusion of these costs is likely to lend much weight to strategies that can safely reduce antimicrobial consumption. In this analysis this would have supported the use of testing for dengue and the use of the CRP test, but less so for the scrub typhus test where overall antimicrobial consumption was either similar to or higher than current practice.

## Conclusion

Pathogen-specific testing can improve the management of non-malarial fevers but their utility and cost-effectiveness is highly sensitive to contextual factors such as heterogeneity of fever aetiologies and pre-existing prescription rates. RDTs that target bacterial infections are more likely to offer tangible direct health benefits but such RDTs are currently not widely available and their accuracies are often limited. Tests for viral infections are available and accurate and can offer some advantages in terms of reducing unnecessary antibiotic prescribing, patient reassurance and surveillance for emerging epidemics, but are less likely to be cost-effective in terms of direct health benefits to the patient. This is a particularly pertinent point where health expenditure is largely paid for by patients out-of-pocket. Testing for biomarkers of host response offers considerable advantages in identifying patients that require antibiotics, but fully materialising these benefits requires appropriate empirical treatment guidelines. Biomarker testing can also reduce the unnecessary use of antibiotics in viral infection, although for CRP considerable over-treatment is likely to remain a limitation due to frequent elevation in non-bacterial infections.
